# Conflicts of interest disclosure policies among Chinese medical journals: A cross-sectional study

**DOI:** 10.1371/journal.pone.0219564

**Published:** 2019-07-09

**Authors:** Jiayi Zhu, Ji Sun

**Affiliations:** 1 International Journal of Blood Transfusion and Hematology, Key Laboratory of Birth Defects and Related Diseases of Women and Children of the Ministry of Education, West China Second University Hospital, Sichuan University, Chengdu, Sichuan, China; 2 Chinese Journal of Obstetrics & Gynecology and Pediatrics (Electronic Edition), West China Second University Hospital, Sichuan University, Chengdu, Sichuan, China; Central South University, The Third Xiang Ya Hospital, CHINA

## Abstract

**Importance:**

Conflicts of interest (COI) disclosure policies are critical to enhancing the integrity of research. However, it is unclear how Chinese medical journals interpret and enforce such policies.

**Objectives:**

The goal of this investigation is to determine the current status of COI disclosure policy enforcement in Chinese medical journals and to promote comprehensive COI policies.

**Methods:**

In this cross-sectional study conducted from September 1^st^ to October 29^th^ 2017, journal instructions, websites and print issues of journals indexed by the Core Journals of China (version 2014), in the medical and health sector, were reviewed to identify whether COI disclosure policies existed and how complete these policies were.

**Results:**

Of 248 eligible journals, 78 (31%) mentioned COI policies; 9 (4%) applied standardized disclosure forms; 18 (7%) required disclosure statements in articles; 4 (2%) mentioned policy bases; none validated disclosed COIs; 2 (1%) mentioned how they dealt with breaches; 18 (7%) involved the management of disclosed COIs; and 62 (25%) and 55 (22%) noted financial and nonfinancial COIs, respectively. Seventy-eight journals (31%) mentioned COIs in research and authors’ obligation towards disclosure; 2 (1%) and 6 (2%) mentioned family members’ and institutional COIs, respectively. Twenty-two and 11 journals mentioned at least one form of financial and nonfinancial COI type in research, respectively. Seven journals (3%) required disclosure of the source of financial support in research, but no journals mentioned the amount of support. Seven (3%) and 12 (5%) journals mentioned COIs in the editorial process and peer-review, respectively. Clinical journals (45%) paid more attention to COI policies than non-clinical journals.

**Conclusions:**

Approximately one-third of Chinese medical journals had COI policies, and of the journals that mentioned financial COIs most required nonfinancial COIs. However, the extent to which journals implemented COI policies was insufficient. There is a generic lack of standardized disclosure forms and management of COIs in most journals. The subject and details of COIs involved in the editorial and peer-review process received less attention than those in research.

## Introduction

Trustworthiness is central to medical research, especially studies with human applications. Polices exist to ensure the integrity of research and merit of science. Among these polices, the disclosure of conflicts of interest (COI) is an important procedure. The Institution of Medicine defines COIs as “circumstances that create a risk that professional judgments or actions regarding a primary interest will be unduly influenced by a secondary interest” [1]. Potential, or actual, COIs mainly exist in research, editorial and peer-review processes, although the latter two receive less attention. COIs of authors, editors, referees, and other participants arise when their primary and secondary interests contradict each other. Authors’ declarations are key to establishing both their financial and nonfinancial COIs. Editors, referees and other participators should also be cautious about COIs that could bias their opinions. Comprehensive guidelines on COI disclosure have been published and promoted by the International Committee of Medical Journal Editors (ICMJE) and Committee on Publication Ethics (COPE) to enhance reproducibility and transparency and to help participants involved in medical publishing to distribute accurate, unbiased research.

There is anecdotal evidence indicating that authors who have relationships with industry tend to publish more positive results, which may favor the industry [2–4]. Negative events have highlighted the need to safeguard scientific integrity and public health. Stakeholders should make efforts to prevent bias caused by COIs and furthermore to discourage ill-considered relationships resulting in COIs [5].

Academic publishing is a cornerstone of the critical evaluation and dissemination of research findings. In efforts to preserve the transparency and integrity of medical research, the overarching role of medical journals cannot be ignored [6]. Efforts have been made to improve the awareness and engagement of authors, referees and publishing participants in COI disclosure and management. Top medical journals have adopted sophisticated COI policies in efforts to ensure the quality of published papers. From 1988 to 2008, the lack of funding disclosure in papers published in three high-impact medical journals: The Lancet, New England Journal of Medicine, and Journal of American Medical Association, decreased from 35% to 7%, and COI reporting increased from 2% to 84% (p < 0.0001) [7]. A study in 2014 showed that all but one of 117 core clinical journals indexed under Abridged Index Medicus had a COI policy [8].

High-impact medical journals have paid increasing attention to COI disclosure. Nevertheless, the awareness and enforcement of COI disclosure policy in Chinese medical journals remains unclear. This study is the first report of a comprehensive analysis of COI disclosure polices among Chinese medical journals, addressing characteristics and contents of the policies and details of financial and nonfinancial COI disclosure in research, editorial, and peer-review processes. We investigated the current status of COI policies applied by Chinese core journals in the field of medicine and health to provide an indication of how COI policies are read and implemented in China.

## Methods

We conducted a cross-sectional study on journal instructions, journal websites and print issues of Chinese medical journals in the medical and health sector. The presence, completeness and related details of COI disclosure policies were examined.

### Study population

The journals that we examined were indexed by the Core Journals of China (version 2014), and are widely recognized by academic circles in China and have profound influence on Chinese periodicals. In the medical and health sector, there were 250 Chinese-language journals classified under 17 discipline categories. Among these were two journals that had no official websites and received submissions via email; they were excluded from this investigation. Thus, 248 eligible journals were examined. The information was mainly extracted from the journals’ websites, Wanfang Med Online (www.med.wanfangdata.com.cn) and China National Knowledge Infrastructure (CNKI) (www.ckni.net); two large bibliographic databases in China.

### Definitions

In this section we defined the key terms and concepts used in this study.

Conflict of interest (COI): We used the Institution of Medicine’s definition of COIs [1] (see Introduction).

Journals with COI policies: Those journals with any form of COI disclosure policy or statement on their website or in print issues, and which require at least one participant’s disclosure.

Standardized COI disclosure form: A unified form which includes a minimum of a COI specification together with the authors’ statement and signature.

COI characteristics: These should include, but not be limited to: the existence of COI policies, a standardized COI disclosure form, and a COI disclosure statement printed at a specific location in published papers.

COI policy contents: These should include, but not be limited to: the basis of COI policies, coverage of financial and nonfinancial COIs, COI disclosure relating to the conduct of research, the editorial process, and the peer-review process, as well as how to authenticate disclosed COIs, how to deal with breaches of the policy, and how COIs are managed.

Financial COIs: Any financial interests and relationships involved in the research and the article to be published. Financial COI types should include specific items such as grants, personal fees, indirect financial support, stock shares, employment and others. The source and amount of financial support should also be specified.

Nonfinancial COIs: Any other COIs apart from financial ones. Nonfinancial COI types should include, but not be limited to, items such as intellectual COIs, relationships, academic competition, beliefs.

Research COIs: Any secondary interests that would influence the integrity of research.

Editorial and the peer-review COIs: Those COIs that would compromise any opinion of participants involved in these key roles of the publication process.

Management of disclosed COIs: The systems for evaluating and handling of disclosed COIs.

COI disclosure subjects: These include authors/editors/referees, family members, institutions and other subjects.

### Data collection

#### General information collection

The impact factor (IF) of each journal was collected from CNKI. General journal characteristics, such as sponsors and assisting organizations, were abstracted from CNKI, Wanfang Med Online as well as the journals’ websites.

#### COI policy-related information collection

To obtain comprehensive data, we gathered data from both the aforementioned databases and the journals’ websites. Journal instructions, instructions for authors published in issue 1, 2017, or the latest available instructions were retrieved and downloaded from the journals’ website and CNKI or Wanfang Med Online. We also searched and copied from each journal’s website any standardized COI disclosure form and information relevant to COI disclosure requirements. As research articles were more likely to involve COIs, the first two original research articles published in issue 1 of 2017, for each journal, were examined to determine whether a COI disclosure statement was present. If there were insufficient research articles, articles from other article types were considered for inclusion. Two articles from every journal were downloaded from the aforementioned databases or journals’ websites. We examined 496 articles in total. All data were collected from September 1^st^ to October 29^th^ 2017.

### Data extraction

To identify all text related to COI, we searched all electronic material for the phrases “conflicts of interest”, “interests”, “relationships”, “conflicts”, “funding”, “support”, “disclosure”, “statement” in Chinese as well as English, as several journals also presented journal instructions and relevant policies in English. We extracted every policy which was described and which asked for disclosure on secondary interests and the relationships of authors, editors, referees, and industries, which could affect the integrity of the research or the quality of articles to be submitted for publication, and anything that could influence the participants’ judgement. We examined the presence and content of any journal instructions, instructions for authors, standardized COI disclosure forms, published article texts and COI-related information to identify whether they contained the aforementioned characteristics, including contents of COI disclosure policies, and types of COIs. Two researchers extracted all data independently using a standardized form. Disputes were settled by reasoned discussion based on agreed definitions. There was high agreement between the researchers extracting data (kappa = 0.9522; 95% CI: 0.9365 to 0.9679).

### Statistical analysis

As they are dictated by different sponsors and academic cultures, COI disclosure policies would be expected to vary across different discipline categories. To examine COI disclosure policy enforcement in different medical fields, we analyzed data in different discipline categories separately. We counted the number and calculated the percentage of journals with different sponsors and assisting organizations. The median impact factor (IF) of journals was calculated and presented as the median together with the interquartile range [IQR]. The number of journals with COI policies, different COI characteristics, specific COI content items, and various COI types were counted, and percentages calculated. Journals with unusual features were analyzed separately.

Statistical analyses were performed using OpenEpi (Version 3: http://www.openepi.com/Menu/OE_Menu.htm). The inter-rater agreement was assessed using Cohen’s kappa coefficient. For journals with IF ≥ median IF, those mentioned COI policies, financial COIs, nonfinancial COIs, COIs in research, editorial process, and peer-review process, were compared with those with IF < median IF by Chi-squared or Fisher’s exact tests. Since the number of journals in the discipline categories of clinical medicine, internal medicine, surgery, obstetrics and gynecology, pediatrics, oncology, neurology and psychiatry, dermatology and venereology, otolaryngology, ophthalmology, and stomatology were small, and all disciplines were under ‘clinical medicine’, we pooled these data into a new category of ‘comprehensive clinical medicine’. The difference in presence of COI policies among journals in different categories were also tested using chi-squared. The threshold level for statistical significance was set at p < 0.05. Multiple comparisons between journals with COI policies under categories of comprehensive medicine and health care, preventive medicine and hygiene, traditional Chinese medicine, preclinical medicine, comprehensive clinical medicine, special medicine, and pharmacy were applied using Chi-squared or Fisher’s exact tests. To take into account multiple testing, we used a Bonferroni corrected P value of < 0.002 (0.05 divided by 21 comparisons) as significance threshold in the instance of the multiple comparisons.

## Results

### General characteristics of journals

When reviewing journal sponsors it was found that for 42 journals (17%) the sponsors were universities/colleges, for 17 journals (7%) it was medical institutions (including research facilities, medical information centers, centers of disease control, etc.), for 86 journals (35%) it was medical associations, for 10 journals (4%) it was hospitals, and for 3 journals (1%) it was publishing houses. Ninety journals (36%) were co-sponsored by at least two entities. Forty-three journals (17%) were assisted by other entities. The median IF of all journals was 1.039 [IQR = 0.573].

### Characteristics and contents of conflicts of interest disclosure policies among journals

Of 248 eligible journals, 78 (31%) mentioned COI policies; 9 (4%) applied standardized disclosure forms; and 18 (7%) required disclosure statements in articles’ footnotes or at the end of the text. Of 496 articles examined, just 34 (7%) included a COI disclosure statement (Table 1). There was a statistically significant difference among journals with COI policies under categories of comprehensive medicine and health care (7 of 37 journals, 19%), preventive medicine and hygiene (7 of 27 journals, 26%), traditional Chinese medicine (1 of 19 journals, 5%), preclinical medicine (6 of 24 journals, 25%), comprehensive clinical medicine (52 of 116 journals, 45%), special medicine (4 of 10 journals, 40%), and pharmacy (1 of 15 journals, 7%) (p = 0.001, chi-square test). In multiple comparisons, a statistically significant difference was found in journals with COI policies between categories of traditional Chinese medicine and comprehensive clinical medicine (p = 0.001, Chi-square test; Bonferroni threshold: p < 0.002). There were more journals with COI policies in categories of comprehensive clinical medicine compared with comprehensive medicine (p = 0.005, Chi-square test) or pharmacy (p = 0.005, Chi-square test), but did not meet the prespecified Bonferroni threshold of p < 0.002. Whereas, the remaining 18 cases of pairwise comparisons between different disciplines of journals with COI policies showed no statistically significant difference. We noticed that 60 journals with COI disclosure policies had no disclosure statements in the published articles, while four journals with statements in print issues had no policies specified in the journal instructions or on their websites.

**Table 1:** Characteristics of COI disclosure policies among journals [n (%)].

Categories	n	COI policy	Standardized COI disclosure form	COI disclosure statement in article
Comprehensive medicine and health care	37	7 (19)	1 (3)	1 (3)
Preventive medicine and hygiene	27	7 (26)	2 (7)	4 (15)
Traditional Chinese medicine	19	1 (5)	0 (0)	0 (0)
Preclinical medicine	24	6 (25)	0 (0)	2 (8) ^a^
Clinical medicine	19	8 (42)	1 (5)	1 (5)
Internal medicine	24	11 (46)	1 (4)	3 (12)
Surgery	26	12 (46)	0 (0)	0 (0)
Obstetrics and gynecology	6	1 (17)	0 (0)	0 (0)
Pediatrics	6	3 (50)	0 (0)	0 (0)
Oncology	9	4 (44)	0 (0)	1 (11)
Neurology and psychiatry	9	6 (67)	0 (0)	2 (22)
Dermatology and venereology	3	1 (33)	0 (0)	0 (0)
Otolaryngology	4	1 (25)	0 (0)	0 (0)
Ophthalmology	5	3 (60)	1 (20)	0 (0)
Stomatology	5	2 (40)	1 (20)	1 (20)
Special medicine	10	4 (40)	2 (20)	3 (30)
Pharmacy	15	1 (7)	0 (0)	0 (0)
Total	248	78 (31)	9 (4)	18 (7)

Abbreviation: COIs, conflicts of interest.

^a^ In two journals, under preclinical medicine, only one out of two articles had COI disclosure statements.

Four journals (2%) mentioned the basis of their COI policies, and the bases were the guidelines of ICMJE or COPE; 62 (25%) and 55 (22%) journals noted financial and nonfinancial COIs specifically; 78 (31%), 7 (3%), and 12 (5%) journals highlighted COIs in research, editorial, and peer-review processes, respectively; no journal had any procedures to authenticate disclosed COIs; 2 (1%) journals mentioned how to deal with COI policy breaches; and 18 (7%) journals had policies to manage disclosed COIs. In several disciplines, most of the aforementioned contents of COI policies were not mentioned (Table 2). Of journals with IF ≥ 1.039, the presence of COI policies, financial COIs, nonfinancial COIs, COIs in research, editorial process, and peer-review process, did not differ from those with IF < 1.039, respectively (p > 0.05, Chi-square test or Fisher’s exact test) (S1 Table). It is worth noting that although the percentage of journals that required COI disclosure in research was low, 234 (94%) journals did require a disclosure related to research funding.

**Table 2:** Contents of COI policies among journals [n (%)].

Categories	n	COI policies basis ^a^	Financial COI	Nonfinancial COI	COI in research	COI in editorial process	COI in review process	Authentication of disclosed COI	Penalties for breaches	Management of disclosed COI ^b^
Comprehensive medicine and health care	37	2 (5)	7 (19)	7 (19)	7 (19)	3 (8)	5 (14)	0 (0)	0 (0)	6 (16)
Preventive medicine and hygiene	27	1 (4)	4 (15)	4 (15)	7 (26)	1 (4)	1 (4)	0 (0)	0 (0)	2 (7)
Traditional Chinese medicine	19	0 (0)	1 (5)	1 (5)	1 (5)	1 (5)	1 (5)	0 (0)	0 (0)	1 (5)
Preclinical medicine	24	1 (4)	3 (12)	3 (12)	6 (25)	1 (4)	1 (4)	0 (0)	0 (0)	0 (0)
Clinical medicine	19	0 (0)	6 (32)	5 (26)	8 (42)	0 (0)	0 (0)	0 (0)	1 (5)	2 (11)
Internal medicine	24	0 (0)	11 (46)	11 (46)	11 (46)	0 (0)	0 (0)	0 (0)	0 (0)	1 (4)
Surgery	26	0 (0)	11 (42)	11 (42)	12 (46)	0 (0)	0 (0)	0 (0)	0 (0)	0 (0)
Obstetrics and gynecology	6	0 (0)	0 (0)	0 (0)	1 (17)	0 (0)	0 (0)	0 (0)	0 (0)	0 (0)
Pediatrics	6	0 (0)	2 (33)	2 (33)	3 (50)	1 (17)	1 (17)	0 (0)	0 (0)	1 (17)
Oncology	9	0(0)	4 (44)	1 (11)	4 (44)	0 (0)	2 (22)	0 (0)	1 (11)	2 (22)
Neurology and psychiatry	9	0(0)	3 (33)	3 (33)	6 (67)	0 (0)	0 (0)	0 (0)	0 (0)	0 (0)
Dermatology and venereology	3	0 (0)	1 (33)	1 (33)	1 (33)	0 (0)	0 (0)	0 (0)	0 (0)	0 (0)
Otolaryngology	4	0 (0)	0 (0)	0 (0)	1 (25)	0 (0)	0 (0)	0 (0)	0 (0)	0 (0)
Ophthalmology	5	0 (0)	3 (60)	2 (40)	3 (60)	0 (0)	0 (0)	0 (0)	0 (0)	1 (20)
Stomatology	5	0 (0)	2 (40)	2 (40)	2 (40)	0 (0)	0 (0)	0 (0)	0 (0)	0 (0)
Special medicine	10	0 (0)	4 (40)	2 (20)	4 (40)	0 (0)	0 (0)	0 (0)	0 (0)	2 (20)
Pharmacy	15	0 (0)	0 (0)	0 (0)	1 (7)	0 (0)	1 (7)	0 (0)	0 (0)	0 (0)
Total	248	4 (2)	62 (25)	55 (22)	78 (31)	7 (3)	12 (5)	0 (0)	2 (1)	18 (7)

Abbreviation: COIs, conflicts of interest.

^a^ Policy basis included ICMJE or COPE.

^b^ Management of disclosed COIs included avoiding referees and editors with COIs; all authors need to declare that no COI existed, and papers with COIs may be rejected.

### Specific items of COIs in research

All of the 78 journals (31%) that mentioned COI policies pointed out the authors’ COIs; 2 (1%) mentioned that it was necessary to disclose family members’ COIs; 6 (2%) pointed out institutional COIs; and no other COI subject was mentioned. Of 17 discipline categories of journal 15 did not mention family members’ COIs and 12 did not mention institutional COIs (Table 3).

**Table 3:** COI subjects involved in research [n (%)].

Categories	n	Author	Family member	Institution	Other	Unspecified
Comprehensive medicine and health care	37	7 (19)	1 (3)	0 (0)	0 (0)	30 (81)
Preventive medicine and hygiene	27	7 (26)	1 (4)	1 (4)	0 (0)	20 (74)
Traditional Chinese medicine	19	1 (5)	0 (0)	0 (0)	0 (0)	18 (95)
Preclinical medicine	24	6 (25)	0 (0)	0 (0)	0 (0)	18 ((75)
Clinical medicine	19	8 (42)	0 (0)	1 (5)	0 (0)	11 (58)
Internal medicine	24	11 (46)	0 (0)	1 (4)	0 (0)	13 (54)
Surgery	26	12 (46)	0 (0)	0 (0)	0 (0)	14 (54)
Obstetrics and gynecology	6	1 (17)	0 (0)	0 (0)	0 (0)	5 (83)
Pediatrics	6	3 (50)	0 (0)	0 (0)	0 (0)	3 (50)
Oncology	9	4 (44)	0 (0)	0 (0)	0 (0)	5 (56)
Neurology and psychiatry	9	6 (67)	0 (0)	0 (0)	0 (0)	3 (33)
Dermatology and venereology	3	1 (33)	0 (0)	0 (0)	0 (0)	2 (67)
Otolaryngology	4	1 (25)	0 (0)	0 (0)	0 (0)	3 (75)
Ophthalmology	5	3 (60)	0 (0)	1 (20)	0 (0)	2 (40)
Stomatology	5	2 (40)	0 (0)	0 (0)	0 (0)	3 (60)
Special medicine	10	4 (40)	0 (0)	2 (20)	0 (0)	6 (60)
Pharmacy	15	1 (7)	0 (0)	0 (0)	0 (0)	14 (93)
Total	248	78 (31)	2 (1)	6 (2)	0 (0)	170 (69)

Abbreviation: COIs, conflicts of interest.

Financial COIs mentioned relating to research covered grants (n = 22, 9%), personal fees (n = 13, 5%), indirect financial support (n = 3, 1%), stock shares (n = 10, 4%), employment (n = 14, 6%), and other types (n = 13, 5%). Other types included expert testimony, board membership, patent application/registration, and benefit-based relationships. Only seven journals (3%) under six discipline categories noted that the source of financial support should be disclosed, but none mentioned the amount (Table 4). Nonfinancial COIs relating to research covered intellectual COIs (n = 4, 2%), personal relationships (n = 7, 3%), academic competition (n = 5, 2%), belief (n = 1, 0%) and other types, i.e., politics (n = 2, 1%) (Table 5).

**Table 4:** Specific items of financial COIs in research [n (%)].

Categories	n	Financial COI types							Source	Amount	Unspecified
		Grant	Personal fee	Indirect financial support	Stock share	Employment	Other ^b^	Unspecified			
Comprehensive medicine and health care	37	3 (8)	1 (3)	1 (3)	1 (3)	1 (3)	1(3)	34 (92)	1 (3)	0 (0)	36 (97)
Preventive medicine and hygiene	27	3 (11)	2 (7)	1 (4)	2 (7)	2 (7)	2 (7)	24 (89)	1 (4)	0 (0)	26 (96)
Traditional Chinese medicine	19	1 (5)	0 (0)	0 (0)	0 (0)	0 (0)	0 (0)	18 (95)	0 (0)	0 (0)	19 (100)
Preclinical medicine	24	1 (4)	0 (0)	0 (0)	0 (0)	0 (0)	0 (0)	23 (96)	0 (0)	0 (0)	24 (100)
Clinical medicine	19	4 (21)	4 (21)	0 (0)	1 (5)	4 (21) ^c^	2 (11)	15 (79)	1 (5)	0 (0)	18 (95)
Internal medicine	24	1 (4)	1 (4)	0 (0)	1 (4)	1 (4)	1 (4)	23 (96)	1 (4)	0 (0)	23 (96)
Oncology	9	2 (22)	1 (11)	0 (0)	1 (11)	1 (11)	2 (22)	7 (78)	0 (0)	0 (0)	9 (100)
Ophthalmology	5	2 (40)	1 (20)	0 (0)	1 (20)	1 (20)	1 (20)	3 (60)	1 (20)	0 (0)	4 (80)
Stomatology	5	2 (40)	1 (20)	1 (20)	1 (20)	2 (40)	2 (40)	3 (60)	0 (0)	0 (0)	5 (100)
Special medicine	10	2 (20)	2 (20)	0 (0)	2 (20)	2 (20) ^c^	2 (20)	8 (80)	2 (20)	0 (0)	8 (80)
Pharmacy	15	1 (7)	0 (0)	0 (0)	0 (0)	0 (0)	0 (0)	14 (93)	0 (0)	0 (0)	15 (100)
Total ^a^	248	22 (9)	13 (5)	3 (1)	10 (4)	14 (6)	13 (5)	226 (91)	7 (3)	0 (0)	241 (97)

Abbreviation: COIs, conflicts of interest.

^a^ All journals under the remaining six discipline categories specified no financial COIs involved in research.

^b^ Other specific items included expert testimony, board membership, patent application/registration, and benefit-based relationships.

^c^ One journal in clinical medicine and one in special medicine mentioned a minimum of three years of employment that needed to be declared.

**Table 5:** Specific items of nonfinancial COIs in research [n (%)].

Categories	n	Intellectual COI	Personal relationship	Academic competition	Belief	Other ^b^	Unspecified
Comprehensive medicine and health care	37	2 (5)	1 (3)	1 (3)	0 (0)	1 (3)	35 (95)
Preventive medicine and hygiene	27	1 (4)	1 (4)	1 (4)	0 (0)	0 (0)	26 (96)
Preclinical medicine	24	1 (4)	0 (0)	0 (0)	0 (0)	0 (0)	23 (96)
Clinical medicine	19	0 (0)	0 (0)	2 (11)	0 (0)	0 (0)	17 (89)
Oncology	9	0 (0)	1 (11)	0 (0)	0 (0)	0 (0)	8 (89)
Ophthalmology	5	0 (0)	1 (20)	0 (0)	0 (0)	0 (0)	4 (80)
Stomatology	5	0 (0)	1 (20)	1 (20)	1 (20)	1 (20)	3 (60)
Pharmacy	15	0 (0)	1 (7)	0 (0)	0 (0)	0 (0)	14 (93)
Total ^a^	248	4 (2)	7 (3)	5 (2)	1 (0)	2 (1)	237 (96)

Abbreviation: COIs, conflicts of interest.

^a^ All journals under the remaining nine discipline categories specified no nonfinancial COIs involved in research.

^b^ Other specific items included politics.

### Specific items of COIs applying to the editorial process

Seven journals (3%) pointed out that editors should disclose COIs; 2 (1%) mentioned family members’ obligation; none pointed out periodical press’ institutional COIs; and 2 (1%) pointed out other subjects, such as invited editors and periodical clerks. Of 17 discipline categories of journal 12 did not mention editors’ obligation to disclose COIs and 15 did not mention family members’ obligation to disclose COIs in editorial process (S2 Table).

One journal in comprehensive medicine and health care (3%) and one in preventive medicine and hygiene (4%) mentioned financial COI types in the editorial process (n = 2, 1%), including personal fees, indirect financial support, stock shares, employment, and other types, such as expert testimony. No journal noted that the source and amount of financial support in the editorial process should be disclosed. The nonfinancial COI types mentioned in the editorial process were intellectual COIs (n = 4, 2%), personal relationships (n = 6, 2%), academic competition (n = 3, 1%), and other types, such as politics (n = 1, 0%). No journal mentioned belief as a nonfinancial COI type. The majority of journals (n = 242, 98%) did not specify nonfinancial COI types. Under 13 discipline categories, no journal noted any type of nonfinancial COI (S3 Table).

### Specific items of COIs in the peer-review process

Eight journals (3%) pointed out that referees should disclose COIs; only 2 (1%) mentioned family members’; and none pointed out referees’ institution’s COIs and other COI subjects in peer-review. Of 17 discipline categories of journal 11 did not mention referees’ obligation to disclose COIs and 15 did not mention that COIs of family members should be disclosed in the peer-review process (S4 Table).

One journal in comprehensive medicine and health care (3%) and one in preventive medicine and hygiene (4%) mentioned financial COI types in the peer-review process (n = 2, 1%), including personal fees, indirect financial support, stock shares, employment, and other types, such as expert testimony. No journals noted that the source and amount of financial support in peer-review should be disclosed. The nonfinancial COI types mentioned in the peer-review process were intellectual COIs (n = 3, 1%), personal relationships (n = 4, 2%), academic competition (n = 3, 1%), and other types, such as politics (n = 1, 0%). No journal mentioned belief as a nonfinancial COI type. Under 14 discipline categories, no journal pointed out any nonfinancial COI type (S5 Table).

## Discussion

Conscious or unconscious bias caused by COIs can compromise the public’s confidence in scientific research, and even cloud the truth, ultimately jeopardizing patient welfare [1]. Medical science has made dramatic contributions to human health and well-being, but the conscious or unconscious bias caused by COIs could unduly compromise further progress. A core goal of medical science has always been to guarantee public trust in medicine. Sophisticated policies established by ICMJE and COPE are aimed to ensure the proper declaration and interpretation of COIs. However, ensuring adequate reporting of all sources of COIs is becoming increasingly challenging as a result of the growing complexity of funding mechanisms and academic culture [9]. One way is to enhance participants’ awareness of COIs but also to not ignore medical journals’ critical role as guardians of propriety and transparency [10]. Leading journals are adopting and promoting COI disclosure policies as part of fulfilling this responsibility [11, 12].

An increasing number of domestic Chinese medical associations and journals’ affiliations and sponsors are recognizing the importance of publication ethics; adopting polices and guidelines from international organizations and aiming to promote policy enforcement in China. The published literature addressing this issue supports this drive. In this context, we present a cross-sectional study of Chinese medical journals, aiming to investigate the current status of COI disclosure policy enforcement, and reflect how COIs are currently disclosed and recognized.

We found 248 eligible medical journals in which 78 (31%) mentioned COI policies. On the other hand, journals cited by Abridged Index Medicus presented sound COI policies [8]. Furthermore, a study published in 2011 showed that of 52 oncology journals listed in the Thomson Institute for Scientific Information, 51 (98%) identified COI disclosures, and 31 (61%) included disclosure statements [12]. In our investigation, only one out of nine oncology journals had COI disclosure statements. While a cross-sectional study carried out in 2012 showed that 9 (90%) Indian and 25 (92.5%) British dental journals required COI declaration [13]. Only 2 out of 5 stomatology journals in our study managed to do so. Compared with these English-language medical journals, some even being reported from more than a decade ago, Chinese medical journals left much to be desired in adherence to guidelines of COI disclosure policies, despite four journals claiming that their COI policies were based on ICMJE or COPE guidelines. Nevertheless, clinical journals that were more likely to be linked with industry generally paid more attention to COI policies (45%).

It is worth noting that 60 journals mentioned COI policies but had no statement in print, and articles with COI statements were published in four journals that did not mention COI policies. This phenomenon was also observed in another study [14]. On the other hand, in two journals, only one of two articles that were examined included a COI declaration statement. This might suggest that the policies were implemented arbitrarily or that authors might be reluctant to disclose COIs publicly. In this circumstance, medical journals should not only specify COI policies but also define them. Authors should be provided with clear guidance on understanding and managing COIs to enhance their engagement with COI disclosure policies.

A sound COI disclosure policy should specify details such as subjects, financial and nonfinancial COIs, and their types. We found that 78 journals (31%) stated that authors should disclose COIs; 2 (1%) and 6 (2%) journals mentioned family members and institutional COIs, respectively. Sixty-two journals (25%) noted financial COIs, and 22 (9%) specified at least one form of financial COI type. Only seven journals (3%) noted that the source of financial support should be disclosed, but no journals mentioned the amount. Fifty-five journals (22%) noted nonfinancial COIs, and 11 (4%) specified nonfinancial COI types. Compared with individual COIs, institutional COIs were less frequently required to be reported. Fortunately, of the journals that mentioned financial COIs, most also pointed out nonfinancial COIs. Comparing periodicals under other indices, in 2014, all 117 core clinical journals indexed under Abridged Index Medicus required the disclosure of financial COIs regarding authors, but only a minority required the disclosure of family members’ (35%) or authors' institution’s (29%) COIs; 57% required nonfinancial COI types, of which two (3%) referred to intellectual COIs [8]. Of 399 biomedical journals of the JCR in 2011, 358 (89.7%) required financial COI disclosure and 280 (70.2%) required nonfinancial COI disclosures [14]. This study also highlighted that clinical journals paid more attention to COI policies than those in fundamental disciplines [14], and this result was consistent with our findings, although it was not statistically significant. Investigation across various fields could provide more insight into the COI disclosure issue [15] and promote journals’ responsibilities to encourage COI declaration policies across different academic cultures.

We also found there was no association between IF and the presence COI policies. This might be due to small differences in journals’ IF, and it might indicate there was a general lack of policies. Nevertheless, it is worth noting that although the number of journals that required COI disclosure in our study was low, 234 journals (94%) did require a disclosure relating to research funding representing a major step forward in the adoption of COI policies.

While journals adopt policies to enhance authors’ understanding of COI declaration, they themselves carry considerable responsibility in implementing policies. They are both supervisors and participators; but only seven journals (3%) noted COIs related to the editorial process. In 2017, a study showed that 34% of 72 health policy and services journals described COIs in the editorial process [16]. In a cross-sectional study of 399 biomedical journals of the JCR in 2011, 155 (38.8%) required editors’ COI disclosures [14]. These studies suggest that there is a generic lack of accessible editorial COI policy among medical journals. COIs involve not only individual but also intuitional behavior. However, institutional COIs have generally received less attention than individual COIs [11]. We found that no journals stated that there should be disclosure of institutional COIs.

Peer-review is a key procedure in scholarly publishing practice; letting referees play a critical role in COI disclosure. Here, 12 journals (5%) mentioned COIs in the peer-review process, and 8 (3%) required referees to disclose COIs. Another survey published in 2015 reported that among 121 Japanese Association of Medical Sciences (JAMS) journals, 60 (49.6%) required editorial committee members to disclose COIs [17]. Encouraging medical journals to adopt comprehensive COI disclosure policies pertaining to the peer-review process, providing guidance on COIs for referees, adopting transparent peer-review, and building reliable referee pools based on disclosed COIs and other critical information might be effective in addressing these issues [18, 19].

Although disclosure has become one of the central approaches to managing COIs, critics contend that it does not serve as a reliable indicator of research credibility, and adopting sophisticated mechanisms to verify the accuracy and completeness of disclosed COIs and to address the breach of COI policies is key [20]. Here, no journal mentioned procedures to authenticate disclosed COIs, and only two journals (1%) mentioned how to deal with any breach of COI disclosure policy. Data were also less than satisfactory among journals in other indexes. As reported in 2017, 72 health policy and services journals had no policy checking the accuracy of disclosed COIs, and only 21% explicitly stated that inaccurate disclosures of COI might result in manuscript rejection [16]. A survey conducted in 2014 reported that, among 121 JAMS journals, only 24 (19.8%) verified the accuracy of declared COIs, and only 33 (27.3%) had clearly stated consequences for violators [17].

We found that 18 journals (7%) had policies to manage disclosed COIs. The proportion was low but still higher than that of the 72 health policy and services journals listed by Web of Science, among which no policy had criteria for COI management [16]. It is noteworthy that of the 18 journals in our study, 11 noted that all authors needed to be able to declare that no COI existed otherwise papers with COIs might be rejected. Some COIs might be unavoidable and it might be too conservative to require a zero tolerance of COIs, such as any link with industry, which may contain risks that could compromise the research results. However, successful collaboration facilitates achievement in medical research and we need to find an appropriate way to manage such collaboration, rather than simply denying it. The severity of COIs depends on the likelihood of undue influence and the seriousness of the potential harm. On the journals’ side, comprehensive criteria for assessing the severity of COIs are in demand. Maharaj [21] proposed a scale to quantify financial COIs on 11 levels. This represents a valuable contribution to the more objective review of research results. COI management should consider the value and scope of secondary interests and the seriousness of potential impacts and make all sources of information available to the public.

Our analysis has certain limitations. We enrolled Chinese core journals, but most Chinese journals were not listed by the index used. Nevertheless, our analysis indicates the main trends in academic publishing in this area. Second, our analysis is based on publicly available information. It could be incomplete as unpublished policies may exist for the processing of manuscript submissions and transcript revisions. Moreover, our study is cross-sectional, looking at a single moment in an ever-changing field but it still represents a contribution to the understanding of current application of COI policies in Chinese medical journals. Despite these flaws, our investigation is an in-depth evaluation of 17 fields of medicine and health, representing its generalizability. One of the aims of this study was to motivate journals to adopt comprehensive policies. Thereby, medical periodicals could become a force in promoting a culture in which COIs are taken seriously by stakeholders. Ultimately, COIs that could compromise primary interests should be discouraged.

Scholarly publishing has the strength of influencing science. To safeguard the integrity of research, journals should recognize the importance of, and explicitly state, comprehensive COI disclosure policies in research, editorial and peer-review processes to promote transparency in rapidly evolving medical areas. Approximately one-third of Chinese medical journals were found to have COI policies, however, the extent to which journals adopted accessible and comprehensive COI policies was insufficient. There was a generic lack of standardized disclosure forms and of the management of COIs in most journals, and the subjects and details of the COIs involved in editorial and peer-review processes received less attention than those in research. Fortunately, in contrast to financial COIs, nonfinancial ones were not neglected in the disclosure policies. There is much to be done, and there may be a long way to go, but medical journals, as one of guardians of medical research quality, should adhere to accepted COI disclosure guidelines, close gaps and loopholes in their COI policies, and make the best decisions for medical publishing practice.

## Supporting information

S1 Table Association of impact factor of journals to COI policies adoption. (PDF)

S2 Table COI subjects involved in editorial process. (PDF)

S3 Table Specific items of nonfinancial COI in editorial process. (PDF)

S4 Table COI subjects involved in peer-review process. (PDF)

S5 Table Specific items of nonfinancial COI in peer-review process. (PDF)
