# Supplementary material for: Conflicts of interest disclosure policies among Chinese medical journals: A cross-sectional study
Source: PLoS One. 2019 Jul 9;14(7):e0219564. doi: 10.1371/journal.pone.0219564 (PMC6615603; doi:10.1371/journal.pone.0219564)
Supplement: S1 Table — (PDF) [file pone.0219564.s001.pdf]

S1 Table. Association of impact factor of journals to COI policy adoption [n (%)].

Impact factor of journals	n	COI policy	Financial COI	Nonfinancial COI	COI in research	COI in editorial process	COI in review process
< 1.039	121	37 (31)	31 (26)	27 (22)	37 (31)	2 (2)	5 (4)
≥ 1.039	127	41 (32)	31 (24)	28 (22)	41 (32)	5 (4)	7 (6)
p		0.772 [a]	0.826 [a]	0.960 [a]	0.772 [a]	0.487 [b]	0.613 [a]

Abbreviation: COIs, conflicts of interest.

[a] Chi-square test was used.

[b] Fisher’s exact test was used with on chi-square value.
