# Supplementary material for: Conflicts of interest disclosure policies among Chinese medical journals: A cross-sectional study
Source: PLoS One. 2019 Jul 9;14(7):e0219564. doi: 10.1371/journal.pone.0219564 (PMC6615603; doi:10.1371/journal.pone.0219564)
Supplement: S2 Table — (PDF) [file pone.0219564.s002.pdf]

S2 Table. COI subjects involved in editorial process [n (%)].

Categories	n	Editor	Family member	Periodical press	Other [b]	Unspecified
Comprehensive medicine and health care	37	3 (8)	1 (3)	0 (0)	1 (3)	34 (92)
Preventive medicine and hygiene	27	1 (4)	1 (4)	0 (0)	0 (0)	26 (96)
Traditional Chinese medicine	19	1 (5)	0 (0)	0 (0)	0 (0)	18 (95)
Preclinical medicine	24	1 (4)	0 (0)	0 (0)	1 (4)	23 (96)
Pediatrics	6	1 (17)	0 (0)	0 (0)	0 (0)	5 (83)
Total [a]	248	7 (3)	2 (1)	0 (0)	2 (1)	241 (97)

Abbreviation: COIs, conflicts of interest.

[a] All journals under the remaining twelve discipline categories specified no COI subjects involved in the editorial process.

[b] Other subjects included invited editors and periodical clerks.
