# Supplementary material for: Conflicts of interest disclosure policies among Chinese medical journals: A cross-sectional study
Source: PLoS One. 2019 Jul 9;14(7):e0219564. doi: 10.1371/journal.pone.0219564 (PMC6615603; doi:10.1371/journal.pone.0219564)
Supplement: S3 Table — (PDF) [file pone.0219564.s003.pdf]

S3 Table. Specific items of nonfinancial COIs in editorial process [n (%)].

**Categories**	**n**	**Intellectual COI**	**Personal relationship**	**Academic competition**	**Belief**	**Other [b]**	**Unspecified**
Comprehensive medicine and health care	37	2 (5)	3 (8)	2 (5)	0 (0)	1 (3)	34 (92)
Preventive medicine and hygiene	27	1 (4)	1 (4)	1 (4)	0 (0)	0 (0)	26 (96)
Traditional Chinese medicine	19	1 (5)	1 (5)	0 (0)	0 (0)	0 (0)	18 (95)
Pediatrics	6	0 (0)	1 (17)	0 (0)	0 (0)	0 (0)	5 (83)
Total [a]	248	4 (2)	6 (2)	3 (1)	0 (0)	1 (0)	242 (98)

Abbreviation: COIs, conflicts of interest.

[a] All journals under the remaining thirteen discipline categories specified no nonfinancial COIs involved in the editorial process.

[b] Other specific types included expert testimony.
